# A Critical Review of Ultra-Short-Term Heart Rate Variability Norms Research

**DOI:** 10.3389/fnins.2020.594880

**Published:** 2020-11-19

**Authors:** Fred Shaffer, Zachary M. Meehan, Christopher L. Zerr

**Affiliations:** ^1^Center for Applied Psychophysiology, Truman State University, Kirksville, MO, United States; ^2^Department of Psychological and Brain Sciences, University of Delaware, Newark, DE, United States; ^3^Department of Psychological and Brain Sciences, Washington University in St. Louis, St. Louis, MO, United States

**Keywords:** biofeedback, Bland–Altman limits of agreement, criterion validity, heart rate variability, norms, Pearson product-moment correlation coefficient, predictive validity, reliability

## Abstract

Heart rate variability (HRV) is the fluctuation in time between successive heartbeats and is defined by interbeat intervals. Researchers have shown that short-term (∼5-min) and long-term (≥24-h) HRV measurements are associated with adaptability, health, mobilization, and use of limited regulatory resources, and performance. Long-term HRV recordings predict health outcomes heart attack, stroke, and all-cause mortality. Despite the prognostic value of long-term HRV assessment, it has not been broadly integrated into mainstream medical care or personal health monitoring. Although short-term HRV measurement does not require ambulatory monitoring and the cost of long-term assessment, it is underutilized in medical care. Among the diverse reasons for the slow adoption of short-term HRV measurement is its prohibitive time cost (∼5 min). Researchers have addressed this issue by investigating the criterion validity of ultra-short-term (UST) HRV measurements of less than 5-min duration compared with short-term recordings. The criterion validity of a method indicates that a novel measurement procedure produces comparable results to a currently validated measurement tool. We evaluated 28 studies that reported UST HRV features with a minimum of 20 participants; of these 17 did not investigate criterion validity and 8 primarily used correlational and/or group difference criteria. The correlational and group difference criteria were insufficient because they did not control for measurement bias. Only three studies used a limits of agreement (LOA) criterion that specified *a priori* an acceptable difference between novel and validated values in absolute units. Whereas the selection of rigorous criterion validity methods is essential, researchers also need to address such issues as acceptable measurement bias and control of artifacts. UST measurements are proxies of proxies. They seek to replace short-term values which, in turn, attempt to estimate long-term metrics. Further adoption of UST HRV measurements requires compelling evidence that these metrics can forecast real-world health or performance outcomes. Furthermore, a single false heartbeat can dramatically alter HRV metrics. UST measurement solutions must automatically edit artifactual interbeat interval values otherwise HRV measurements will be invalid. These are the formidable challenges that must be addressed before HRV monitoring can be accepted for widespread use in medicine and personal health care.

## Introduction

The purpose of this review article is to critically examine the criteria used in studies of ultra-short-term (UST) heart rate variability (HRV) and to identify challenges of criterion, concurrent, and predictive validity, and measurement artifacts.

Section “Heart Rate Variability” explains HRV from the perspectives of the neurovisceral integration mode and vagal tank theory. We underscore that HRV metrics are associated with regulatory capacity and health, providing an indication of how HRV predicts health crises such as fetal distress before the appearance of symptoms or mortality. Further, these metrics describe the correlation between low HRV, disease, and mortality.

Section “Length of the HRV Recording Period” describes long-term, short-term, and UST HRV recordings, and it emphasizes that long-term measurements best predict health outcomes, and provides a description of time domain, frequency domain, and non-linear metrics. We explain that short-term measurements poorly correlate with long-term values, and stress that we cannot use long-term and short-term norms interchangeably. We caution that short-term measurements are proxies of long-term measurements and that their predictive validity is uncertain. Finally, we characterize UST measurements as proxies of proxies and call for research into their predictive validity.

Section “Why Is There Interest in UST HRV Measurements?” discusses the reasons for the limited use in HRV measurements in medicine, the challenges to their integration into routine medical care, the opportunity created by wearable products for consumer HRV monitoring, and the research required before the widespread adoption of HRV metrics in fitness and wellness applications.

Section “Criterion Validity Ensures Measurement Integrity” explains criterion validity, which can be established using the concurrent and predictive validity approaches. These approaches depend on a high-quality criterion that is relevant, reliable, and valid.

Section “UST HRV Research” provides an overview of 28 studies that have reported UST HRV features. We argue that comparison approaches using correlational coefficients, coefficients of determination or regression, and group mean or median comparisons approaches cannot establish criterion validity because they do not control for measurement bias, which is the difference between novel and validated measurements. Section “Correlation Coefficients” explains that although correlation coefficients can identify potential surrogates, they cannot establish criterion validity. Correlations show association but cannot establish equivalence. A proxy measurement can be perfectly correlated with a reference standard measurement while falling outside an acceptable range (e.g., ±10% of the reference standard’s range). Section “Coefficient of Determination or Regression” argues that neither method is appropriate for demonstrating equivalence. The coefficient of determination shares the same limitations as correlation coefficients and use of regression for this purpose violates its underlying statistical assumptions. Section “Group Mean or Median Comparisons” challenges the claim that two methods are comparable if they yield a non-significant group mean or median difference because this does not ensure validity and can be confounded by insufficient statistical power. Lastly, Section “Limits of Agreement (LOA) Solutions” describes how this approach establishes criterion validity when accuracy standards are specified *a priori*.

Section “UST HRV Studies Reporting Limits of Agreement Solutions” summarizes four studies that have reported LOA and compares findings from three reports (Esco and Flatt, 2014; Munoz et al., 2015; Shaffer et al., 2019) that utilized LOA as a selection criterion for valid UST measurements. Finally, Section “Practical Recommendations” outlines four steps for determining the shortest period that can estimate a 300-s measurement.

## Heart Rate Variability

Heart rate and HRV are calculated from the time intervals between successive heartbeats and HRV is associated with executive function, regulatory capacity, and health (Thayer and Lane, 2000; Byrd et al., 2015; Laborde et al., 2017; Mather and Thayer, 2018). *Heart rate*, the number of heart beats per minute (bpm), is an UST (<5 min) metric that is widely used in medicine, performance, and daily fitness assessment using wearables. HRV is the organized fluctuation of time intervals between successive heartbeats defined as interbeat intervals (Shaffer and Ginsberg, 2017; Lehrer et al., 2020). The complexity of a healthy heart rhythm is critical to the maintenance of homeostasis because it provides the flexibility to cope with an uncertain and changing environment (Beckers et al., 2006). “A healthy heart is not a metronome” (Shaffer et al., 2014). From the perspective of the neurovisceral integration model (Thayer and Lane, 2000), increased HRV is associated with improved executive function and may strengthen descending medial prefrontal cortex regulation of emotion (Mather and Thayer, 2018). Laborde et al. (2018) have proposed the *vagal tank theory* as an integrative model of *cardiac vagal control* or vagus nerve regulation of heart rate. Cardiac vagal control indexes how efficiently we mobilize and utilize limited self-regulatory resources during resting, reactivity, and recovery conditions (Laborde et al., 2017). HRV metrics are important because they are associated with regulatory capacity, health, and performance (Shaffer et al., 2014) and can predict morbidity and mortality.

A decline in HRV can signal dangerous health changes and low HRV values are associated with an increased risk of illness and death. HRV reductions precede heart rate changes in conditions of fetal distress (Hon and Lee, 1963) and sensory disturbances in diabetic autonomic neuropathy (Ewing et al., 1976). Low HRV correlates with anxiety (Cohen and Benjamin, 2006), asthma (Kazuma et al., 1997; Lehrer et al., 2004), cardiac arrhythmia, chronic obstructive pulmonary disease (Giardino et al., 2004), depression (Agelink et al., 2002), functional gastrointestinal disorders (Gevirtz, 2013), hypertension, inflammation, myocardial infarction (Bigger et al., 1992; Carney et al., 2007; Berntson et al., 2008), post-traumatic stress disorder (Shah et al., 2013), and sudden infant death (Hon and Lee, 1963). Low HRV also correlates with all-cause mortality (Tsuji et al., 1994; Dekker et al., 1997). For example, low power in the very-low-frequency (VLF) band (0.0033–0.04 Hz) more strongly predicted all-cause mortality (higher *Z*-scores and relative risk) than low-frequency (LF; 0.04–0.15 Hz) and high-frequency (HF; 0.15–0.4 Hz) bands, and is associated with arrhythmic death (Bigger et al., 1992).

## Length of the HRV Recording Period

Heart rate variability recording periods range from under 1 min to over 24 h. *Long-term recordings* (≥24 h) constitute the reference standard for clinical evaluation due to their *predictive validity*, which is the ability to predict future outcomes (Hoenig et al., 2001). For example, 24-h measurements of the standard deviation (SD) of the interbeat intervals of normal sinus beats (SDNN) predict cardiac risk (Task Force of the European Society of Cardiology the North American Society of Pacing Electrophysiology, 1996). Acute myocardial infarction patients with SDNN values under 50 ms are unhealthy, between 50 and 100 ms have compromised health, and over 100 ms are healthy (Kleiger et al., 1987). Acute myocardial infarction patients with SDNN values over 100 ms have been reported to have a 5.3 lower mortality risk at a 31-month mean follow-up than those under 50 ms.

While long-term, short-term (∼5 min), and UST (<5 min) recordings calculate HRV metrics using the same mathematical formulas, they are not interchangeable, reflect different underlying physiological processes, and achieve different predictive powers. HRV in long-term recordings may be attributed to changes in the circadian rhythm, fluctuations in core body temperature and the renin–angiotensin system, and the sleep cycle (Bonaduce et al., 1994; Task Force of the European Society of Cardiology the North American Society of Pacing Electrophysiology, 1996). Long-term recordings monitor cardiorespiratory regulation across diverse situations, physical workloads, and anticipatory central nervous system (CNS) reactions to environmental stimuli. These extended recording periods reveal the sympathetic nervous system (SNS) component of HRV (Grant et al., 2011; Shaffer and Ginsberg, 2017). HRV in short-term recordings is produced by four interdependent sources that operate on a briefer time scale and are defined by: (1) the complex interaction between the sympathetic and parasympathetic branches; (2) respiration-mediated increases and decreases in heart rate via the vagus nerve, termed respiratory sinus arrhythmia (RSA); (3) the baroreceptor reflex that regulates blood pressure using negative feedback; and (4) rhythmic adjustments in blood vessel diameter (Shaffer and Ginsberg, 2017). Short-term values correlate poorly with their long-term counterparts (Fei et al., 1996). Basic research is needed to identify the major HRV generators in UST recordings.

Although long-term, short-term, and UST HRV recordings are characterized using the same time-domain, frequency-domain, and non-linear indices, they differ in predictive power. *Time-domain* metrics calculate the amount of variability in a series of interbeat intervals. *Frequency-domain* measurements compute absolute or relative power distribution across four bands: *ultra-low-frequency* (ULF; ≤0.003 Hz), VLF (0.0033–0.04 Hz), LF (0.004–0.15 Hz), and HF (0.15–0.40 Hz). *Non-linear* indicators measure the interbeat interval time series’ unpredictability (Stein and Reddy, 2005; Table 1). ST recordings achieve lower predictive power than long-term recordings (Bigger et al., 1989; Nolan et al., 1998; Kleiger et al., 2005). To summarize, long-term recordings represent the reference standard for predicting health outcomes. For this reason, long-term and short-term norms cannot be used interchangeably. Short-term values are proxies of long-term values with unknown predictive validity; therefore, UST measurements are proxies of proxies. Basic research is also needed to determine the predictive validity of UST recordings.

**TABLE 1:** Short-Term HRV metrics adapted from Shaffer and Ginsberg (2017) and Shaffer et al. (2019).

**HRV metrics**	**Units**	**Description**
**Time domain**		
Heart rate	1/min	Average heart rate
HRV triangular index (HTI)		Integral of the density of the RR interval histogram divided by its height; together, HTI and RMSSD can distinguish between normal rhythms and arrhythmias
NN	ms	Average of NN intervals
NN50	count	Number of successive RR intervals that differ by more than 50 ms
pNN50	%	Percentage of successive RR intervals that differ by more than 50 ms; associated with HF absolute power and RMSSD
RMSSD	ms	Root mean square of successive RR interval differences; estimates vagal contributions to HRV
SDNN	ms	Standard deviation of NN intervals; strongly associated with ULF, VLF, LF, and total power; vagally-mediated RSA is primary source, especially with slow, paced breathing during ST recording
TINN		Baseline width of the RR interval histogram
**Frequency domain**		
VLF	ms^2^	Absolute power of the very-low-frequency band (0.0033–0.04 Hz)
LF	ms^2^	Absolute power of the low-frequency power (0.04–0.15 Hz)
LFnu	nu	Relative power of the low-frequency band in normal units
HF	ms^2^	High-frequency power (0.15–0.4 Hz)
HFnu	nu	Relative power of the high-frequency band in normal units
LF/HF	%	Ratio of LF-to-HF absolute power
Total	ms^2^	Sum of absolute power in the VLF, LF, and HF bands in ST recordings
**Non-linear**		
ApEn		Approximate entropy, which measures the regularity and complexity of a time series; small values mean signal predictability
D_2_		Correlation dimension, which estimates the minimum number of variables required to construct a model of system dynamics; more variables mean greater time series complexity
DET	%	Recurrence plot analysis determinism
DFα1		Detrended fluctuation analysis, which describes short-term fluctuations; reflects the baroreceptor reflex
DFα2		Detrended fluctuation analysis, which describes long-term fluctuations; reflects regulation of interbeat interval fluctuation
REC	%	Recurrence rate
SampEn		Sample entropy, which measures the regularity and complexity of a time series; like ApEn, small values mean signal predictability
SD1	ms	Poincaré plot standard deviation perpendicular to the line of identity; measures ST HRV and is associated with baroreflex sensitivity (BRS)
SD2	ms	Poincaré plot standard deviation along the line of identity; measures ST and LT HRV and is associated with LF absolute power and BRS
ShanEn		Shannon entropy; measures the average information in a time series; higher values indicate greater uncertainty and irregularity

*Credit: Center for Applied Psychophysiology. Baroreflex sensitivity (BRS), the change in interbeat interval length per unit change in BP and HF absolute power; normal units (nu) are determined by dividing frequency band absolute power by the summed the absolute power of the LF and HF bands; frequency domain, measurements that compute absolute or relative power distribution across four bands: ultra-low-frequency (ULF; ≤0.003 Hz), very-low-frequency (VLF; 0.0033–0.04 Hz), low-frequency (LF; 0.004–0.15 Hz), and high-frequency (HF; 0.15–0.40 Hz); non-linear, indicators that measure the interbeat interval time series’ unpredictability; short-term, measurements ∼ 5 min; time domain, metrics that calculate the amount of variability in a series of interbeat intervals.*

## Why Is There Interest in UST HRV Measurements?

There is a potential role for UST HRV measurements in medical assessment, research involving brief (e.g., <30 s) experimental tasks, and personal wellness assessment once researchers validate their accuracy and predictive power. Despite the availability of short-term normative HRV values for adults (Umetani et al., 1998; Nunan et al., 2010) and elite athletes (Berkoff et al., 2007), HRV is not widely used in medical assessment outside of cardiology and obstetrics. For example, nurses do not routinely monitor HRV as a vital sign during general practice visits. Short-term HRV assessment’s time cost is one of many barriers to its integration in routine medical practice: “…a 5-min HRV assessment is prohibitively long when compared with routine office or home measurements of blood glucose, blood pressure, core body temperature, heart rate, oxygen saturation, and weight” (Shaffer et al., 2019, p. 215). If researchers were to validate the accuracy and predictive power of UST HRV measurements, and provide age- and sex-related normative values, manufacturers could add this modality to widely used instruments like electrocardiographs and pulse oximeters.

Research studies in diverse areas (e.g., clinical and social psychology) may involve brief experimental tasks that require UST HRV measurements. For example, short-term HRV monitoring would be inappropriate for a 30-s task designed to induce frustration. As with medical applications, researchers need to validate the accuracy and meaning of UST HRV measurements.

Consumers increasingly monitor their physiology using dedicated tracking devices and smartwatches that incorporate electrocardiographic (ECG) and photoplethysmographic (PPG) sensors of heart rate and HRV. ECG sensors detect the R-spike and PPG sensors identify the peak of the pulse wave to determine when a heartbeat has occurred (Shaffer et al., 2014). The ECG method is more accurate than PPG during paced breathing (Jan et al., 2019) and when increased sympathetic tone results in vasoconstriction in monitored fingers (Giardino et al., 2002; Schafer and Vagedes, 2013). UST measurements are ideal for these ambulatory fitness and wellness applications if investigators can demonstrate their accuracy under non-stationary and stationary conditions, their predictive validity, and normative values.

## Criterion Validity Ensures Measurement Integrity

*Criterion validity* confirms that test scores accurately estimate scores of validated measures or metrics and depends on the identification of a high-quality criterion (Gulliksen, 1987). Researchers use concurrent and predictive validity approaches to provide evidence of criterion validity. In the *concurrent* approach, investigators obtain test and criterion scores simultaneously (Price, 2018). The UST HRV studies reviewed in this article illustrate this strategy. Here, the test scores are UST and the criterion scores are short-term HRV values. In the *predictive* approach, researchers obtain test scores to estimate future outcomes or performance. The success of both strategies depends on the existence of a *high-quality criterion*, which is relevant, valid, and reliable (Price, 2018). *Relevant* means that we can objectively assess the criterion (e.g., SDNN). *Validity* means that the criterion (e.g., 5-min SDNN) accurately measures the metric of interest (e.g., SDNN). Finally, *reliability* means that criterion scores (e.g., 5-min SDNN values) obtained from the same individuals under identical conditions are consistent. Although valid measures are always reliable, reliable measures are not valid unless they accurately assess a given construct (e.g., SDNN).

## UST HRV Research

We evaluated 28 studies that reported UST HRV features with a minimum of 20 participants (Table 2). Seventeen studies did not investigate criterion validity. Eight studies primarily used correlational and/or group difference criteria to demonstrate the criterion validity of UST (test scores) with respect to short-term values (criterion scores; Thong et al., 2003; Schroeder et al., 2004; McNames and Aboy, 2006; Salahuddin et al., 2007; Li et al., 2009; Nussinovitch et al., 2011; Baek et al., 2015; Brisinda et al., 2015). Correlation coefficients, the coefficient of determination or regression, and group mean or median comparisons are insufficient to establish criterion validity because they do not control for *measurement bias*—the difference between UST and short-term measurements.

**TABLE 2:** Studies that reported UST HRV measurements and their primary criterion validity criteria.

**Did Not Investigate UST Criterion Validity**	
Arza et al. (2005)	Pandey et al. (2016)
Choi and Gutierrez-Osuna (2009)	Papousek et al. (2010)
De Rivecourt et al. (2008)	Pereira et al. (2017)
Hjortskov et al. (2004)	Schubert et al. (2009)
Kim et al. (2008)	Sun et al. (2010)
Kwon et al. (2016)	Wang et al. (2009)
Li et al. (2009)	Wijsman et al. (2011)
Mayya et al. (2015)	Xu et al. (2015)
Nardelli et al. (2018)	
**Correlational and/or Group Difference UST Criterion Validity Criteria**	
Baek et al. (2015)	Munoz et al. (2015)
Brisinda et al. (2015)	Nussinovitch et al. (2011)
Esco and Flatt (2014)	Salahuddin et al. (2007)
Li et al. (2009)	Schroeder et al. (2004)
McNames and Aboy (2006)	Thong et al. (2003)
**Limits of Agreement UST Criterion Validity Criterion**	
Esco and Flatt (2014)	
Munoz et al. (2015)	
Shaffer et al. (2019)	

*Credit: Center for Applied Psychophysiology. Correlational criterion, two methods are equivalent if their values are correlated; concurrent validity, a novel measurement procedure produces comparable results to an already validated measurement tool; HRV, heart rate variability; group difference criterion, two methods are comparable if they yield a non-significant group mean or median difference; limits of agreement criterion, two methods are equivalent if there is an acceptable *a priori* difference between their values in absolute units; UST, ultra-short-term (<5 min).*

### Correlation Coefficients

**FIGURE 1:**
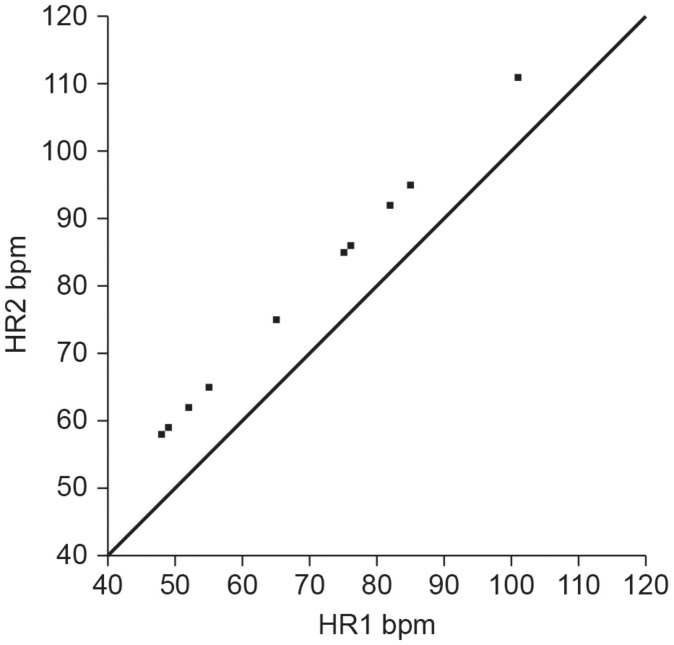
Hypothetical scatterplot of UST and ST heart rates (bpm) depicting a perfect correlation (*r* = 1), but no agreement (points do not fall along the line of equality where *y* = *x*). Credit: Center for Applied Psychophysiology.

Although correlation analysis can help researchers identify potential surrogates, they cannot measure criterion validity (Pecchia et al., 2018). Many researchers make the mistake of applying a correlation coefficient, typically Pearson’s *r*, to conclude that two methods are sufficiently comparable or in agreement. The Pearson *r* quantifies the direction, magnitude, and probability of a linear relationship between two continuous variables, *x* and *y*. The magnitude of the Pearson *r* ranges from −1 to +1 (Devore, 2016). A correlation coefficient, however, is merely a measure of association and does not provide evidence that one method agrees with or is comparable to another method (Altman and Bland, 1983). In fact, it is possible for two methods to have a perfect correlation of *r* = 1 but no agreement or comparability between the measurements (Watson and Petrie, 2010). For example, consider the situation where Method A and Method B both measure heart rate, but only Method A does this accurately. If Method B yields readings that are consistently 10 bpm higher than Method A, they would be perfectly correlated (*r* = 1) but their measurements would disagree by 10 bpm (Figure 1).

**FIGURE 2:**
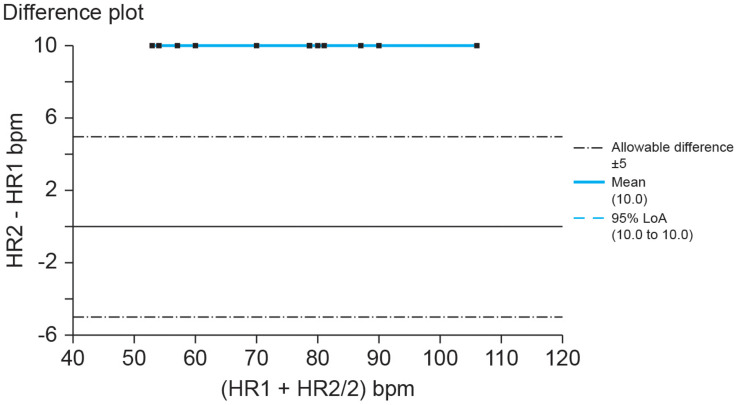
Hypothetical Bland–Altman difference plot of UST and ST heart rates (bpm). Credit: Center for Applied Psychophysiology. The line at 0 represents the line of equality or *y* = *x* (the diagonal line from Figure 1). When measures achieve absolute agreement, they will all fall along that line at 0.

The American National Standards Institute criterion (ANSI/AAMI, 2002) for heart rate accuracy is the larger of ±10% of all values or ±5 bpm. If we set the allowable heart rate difference at ±10% of Method A’s range, Method B would report heart rates far beyond acceptable measurements as shown by a Bland–Altman plot (Figure 2).

Additionally, a significant correlation between two different methods “is generally useless because two methods designed to measure the same quantity will rarely be uncorrelated” (Choudhary and Nagaraja, 2005, p. 218). For these reasons, researchers conclude that a “correlation coefficient … is of no practical use in the statistical analysis of comparison data” (Westgard and Hunt, 1973, p. 53).

### Coefficient of Determination or Regression

Some method comparison studies use the coefficient of determination (*r*^2^) or simple regression analysis to claim two methods are comparable via intercepts or slopes (Bland and Altman, 2003). The *coefficient of determination* estimates the percentage of variability of variable *y* that can predicted by *x*. Denoted as *r*^2^, the coefficient of determination is identical to the square of the Pearson *r* coefficient. For example, a Pearson *r* coefficient of 0.50 corresponds to an *r*^2^ value of 0.25, meaning that 25% of the variability in *y* is accounted for by variability in *x*. The magnitude of *r*^2^ ranges from −1 to +1. *Simple regression analysis* estimates a straight line with a slope (B_1_) and height at which the line crosses the vertical axis (B_0_) to predict the value of *y*, given *x* (Devore, 2016). These measures are also inappropriate for demonstrating agreement. The coefficient of determination estimates the proportion of variance that Method A and Method B share but present the same pitfalls as the correlation coefficient (Zaki et al., 2012). In addition, the coefficient of determination calculates how well a regression equation or model fits the observed data. This is problematic for method comparison studies as measurements from each method are dependent variables, each possessing their own measurement error. Linear regression models make an implicit assumption that some portion of the variance in a dependent variable (Y) is being explained by variance in an independent variable (X). Therefore, a simple linear regression assumes that the procedure measures X without error. This method is not appropriate when comparing two dependent measures and may produce a biased regression coefficient (Altman and Bland, 1983; Hays, 1991). If regression is used, both variables should be treated as possessing measurement error. In these cases, Deming regression (parametric) or Passing–Bablok regression (non-parametric) are more appropriate alternatives (Giavarina, 2015).

Deming regression (Deming, 1943) is a type of total least squares regression that accounts for measurement error in both X and Y variables, as opposed to ordinary least squares regression which merely accounts for error in the dependent variable. Deming regression assumes that errors are independent and normally distributed, but the procedure is sensitive to outliers. Passing–Bablok regression (Passing and Bablok, 1983, 1984) is a robust non-parametric rank method that also accounts for error in both X and Y and produces an unbiased slope estimate by calculating the median of all possible slopes (Linnet, 1993). Passing–Bablok regression is less sensitive to outliers and does not have assumptions about the distribution of errors, but it does require that the two variables measured do not significantly deviate from linearity (Passing and Bablok, 1983).

### Group Mean or Median Comparisons

Another statistical approach misused in method comparison studies is to claim that two methods are comparable if they yield a non-significant group mean or median difference via parametric or non-parametric tests. For example, a *two-sample t-test* is a parametric statistic that evaluates whether the difference between pairs of normally-distributed scores can be explained by chance. A *Kruskal–Wallis test* is a non-parametric procedure that determines whether samples were obtained from a single distribution (Devore, 2016). There are several issues with such an approach. First, the goal of comparing two different methods of measurement is not to have an equivalent overall group agreement (mean or median), but rather that the methods appropriately agree across individual observations. Such logic would imply that having greater measurement error would be more favorable because it decreases the probability of finding a significant difference (Altman and Bland, 1983). Non-significant group differences do not indicate whether two methods agree or have acceptable bias. Second, significance is related to the power and sample size of the study (Zaki et al., 2012), and so a non-significant mean or median difference between two methods could be the result of an underpowered study or one without a large enough sample. Third, because many HRV measures are non-normally distributed, some studies inappropriately use a parametric *t*-test or ANOVA on data that have not been log-transformed or fail to use a non-parametric test instead (Pecchia et al., 2018).

### Limits of Agreement (LOA) Solutions

To overcome the aforementioned issues with analyzing agreement between methods, the authors recommend the use of LOA in Bland–Altman plots (Altman and Bland, 1983; Bland and Altman, 1986). An important caveat is that Bland–Altman plots and LOA do not indicate whether or not the agreement between measures is sufficient. The researcher must decide *a priori* the extent to which two measures must agree for them to be comparable. Although there are industry standards for the accuracy of blood pressure and heart rate measurement (ANSI/AAMI, 2002, 2008), there are no comparable standards for HRV short-term measurements such as SDNN. The degree of precision may depend upon the specific question being asked and may vary by discipline (Giavarina, 2015).

Bland–Altman plots are a graphical approach to assessing the extent to which two methods agree with each other by plotting the difference between the two methods (Method A – Method B) on the *y*-axis against the mean of the two methods ([Method A + Method B]/2) on the *x*-axis. If the two methods agree completely, the mean difference ($\bar{d}$) between them will be zero, and all the points on the Bland–Altman plot would fall along a line of *y* = 0. Because perfect agreement between two methods rarely occurs, the distance between an ideal $\bar{d}$ of zero and the observed $\bar{d}$ is an index of bias. The greater the bias—the distance of $\bar{d}$ from zero—between the two methods, the less the two measures tend to agree. Assuming that the differences are normally distributed, the SD of the differences can then be multiplied by 1.96 and added/subtracted from the mean difference $\bar{d}$. This calculation produces a lower LOA ($\bar{d}$ – 1.96*s*) and an upper LOA ($\bar{d}$ + 1.96*s*), representing the range where 95% of the differences should fall; the lower LOA represents the 2.5th percentile and the upper LOA represents the 97.5th percentile.

Researchers should construct confidence intervals and statistically determine whether the disagreement between the two methods falls within the LOA. They should construct 95% confidence intervals around the mean difference and the lower/upper LOA to take variability into account (Hamilton and Stamey, 2007; Ludbrook, 2010). Next, they should perform a statistical analysis to determine whether the differences between the two methods fall within the appropriate LOA (Giavarina, 2015). Finally, they should follow with an equality test (H_0_: μdifference = 0) such as the Student’s *t*-test. Bland–Altman plots do not require the raw measurements from the two methods to be normally distributed, but the *differences* between the two methods should be normally distributed. Researchers should take appropriate steps if the differences are not normally distributed or the differences are proportional to the size of the measurement (e.g., greater differences between the two methods as the measurements get larger). They can logarithmically transform the raw data or the ratios or percentages ([Method A – Method B]/Mean%) before constructing a Bland–Altman plot. This transformation can provide superior results to plotting a simple difference between the methods against the average (Giavarina, 2015; Hoffman, 2015). In addition to assessing agreement, Bland–Altman plots can also be used to detect outliers (Watson and Petrie, 2010).

## UST HRV Studies That Report Limits of Agreement Solutions

Of the 28 UST HRV studies that we reviewed, four reported LOA plots whether used as a selection criterion or not (Esco and Flatt, 2014; Baek et al., 2015; Munoz et al., 2015; Shaffer et al., 2019) (Table 3).

**TABLE 3:** UST studies that reported limits of agreement adapted from Shaffer and Ginsberg (2017).

**Study, date**	****N****	**Method**	**Position**	**Conditions**	**UST (s)**	**HRV metrics**	**UST criteria**
Baek et al., 2015	467 249 men 218 women	PPG	Sitting	Baseline	10–270	HR, pNN50, RMSSD, SDNN, VLF, LF, HF, LF/HF, Total, LFnu, HFnu	Pearson **r** and non-significant Kruskal–Wallis
Esco and Flatt, 2014	23 men	ECG	Supine	Pre/post-exercise	10, 30, 60	RMSSD	ICC and Bland–Altman
Munoz et al., 2015	3,387 1658 men 1729 women	Portapres^®^	Supine	Baseline	10, 30, 120	RMSSD, SDNN	ICC, Pearson **r**, and Bland–Altman
Shaffer et al. (2019)	38 20 men 18 women	ECG	Sitting	Baseline	10, 20, 30, 60, 90, 120, 180, 240	Table 1	**r** ≥ 0.90 and Bland–Altman LOA ± 5% of the range

*Credit: Center for Applied Psychophysiology. D_2_ (also CD), correlation dimension, which estimates the minimum number of variables required to construct a model of a studied system; DFA α1, detrended fluctuation analysis, which describes short-term fluctuations; DFA α2, detrended fluctuation analysis, which describes long-term fluctuations; ECG, electrocardiogram; HF ms^2^, absolute power of the high frequency band; HF nu, relative power of the high frequency band in normal units; HF peak, highest amplitude frequency in the HF band; HF%, HF power as a percentage of total power; HR, heart rate; HTI, HRV triangular index or integral of the density of the NN interval histogram divided by its height; limits of agreement, criterion that two methods are equivalent if there is an acceptable *a priori* difference between their values in absolute units; LF ms^2^, absolute power of the low frequency band; LF nu, relative power of the low frequency band in normal units; LF peak, highest amplitude frequency in the LF band; LF%, LF power as a percentage of total power; LF/HF, ratio of LF-to-HF power; NN interval, time between adjacent normal heartbeats; nu, normal units calculated by dividing the absolute power for a specific frequency band by the summed absolute power of the LF and HF bands; pNN50, percentage of successive interbeat intervals that differ by more than 50 ms; RMSSD, root mean square of successive R–R interval differences; R–R interval, time between all adjacent heartbeats; SampEn, sample entropy, which measures signal regularity and complexity; SD1, Poincaré plot standard deviation perpendicular to the line of identity; SD2, Poincaré plot standard deviation along the line of identity; SD1/SD2, ratio of SD1 to SD2 that measures the unpredictability of the R–R time series and autonomic balance under appropriate monitoring conditions; SDNN, standard deviation of NN intervals; TINN, triangular interpolation of the R–R interval histogram or baseline width of the RR interval histogram; total power, sum of power (ms^2^) in VLF, LF, and HF bands; UST, ultra-short-term (<5 min).*

Baek et al. (2015) obtained resting PPG measurements from 467 healthy participants (249 men and 218 women; aged 8–69 years). They compared 10-, 20-, 30-, 60-, 90-, 180-, 210-, 240-, and 270-s values with 300-s measurements. Their criteria for selecting the shortest UST period were a significant Pearson *r* and non-significant (*p* > 0.05) Kruskal–Wallis statistic. Although they illustrated their results with Bland–Altman plots (mean difference ± 1.96 *SD*), the authors did not use them to draw conclusions.

Esco and Flatt (2014) acquired ECG measurements from 23 male collegiate athletes (aged 19–21 years) for 10 min while supine before a treadmill test and for 30 min post-exercise. They analyzed the last 5 min of each rest period and compared log-transformed 10-, 30-, and 60-s with 300-s root mean square of the successive differences (RMSSD) values. They compared intra-class correlations (ICCs) and Bland–Altman plots (mean difference ± 1.96 *SD*) across the three UST periods and concluded that that 60 s yielded the largest ICC and most stringent LOA. Whereas the ICC test identified 60 s as a potential surrogate, a Bland–Altman plot confirmed its criterion validity with respect to 300-s RMSSD measurements.

Munoz et al. (2015) recorded beat-to-beat middle finger pressure using a Portapres^®^ device from 3387 participants (1660 men and 1727 women; aged 44–63 years) in the Prevention of Renal and Vascular End-Stage Disease study. They obtained recordings over a 15-min period while resting in the supine position. The authors analyzed the last 4–5 min of data that exhibited a stationarity pattern and compared the log-transformed 10-, 30-, and 120-s with 300-s RMSSD and SDNN values. They compared ICC, Pearson *r* values, and Bland–Altman plots across the three UST periods. The authors concluded that a minimum of 10 s was required to measure RMSSD and 30 s to calculate SDNN.

Shaffer et al. (2019) obtained 5-min EEG recordings from 38 healthy undergraduates (20 men and 18 women; aged 18–23 years) while sitting upright under resting conditions with their eyes open. They acquired 10-, 20-, 30-, 60-, 90-, 120-, 180-, and 240-s epochs from the 5-min recordings. Following manual removal of artifacts, they calculated the time domain, frequency domain, and non-linear HRV metrics outlined in Table 1. The authors identified potential surrogates using a Pearson *r* with a conservative criterion (*r* ≥ 0.90). They applied Bland–Altman’s LOA technique using an allowable difference of ±5% of the range of the 5-min value and a Student’s *t*-test to confirm the equality of UST and ST values. The results of LOA analyses are summarized in Table 4. These findings were consistent with Esco and Flatt (2014) who also reported that a time interval of 60 s was required to estimate 5-min RMSSD. However, the finding that a 60-s sample is required to measure RMSSD and SDNN was inconsistent with the study by Munoz et al. (2015) who reported minimum periods of 10 and 30 s, respectively. This disagreement may have been due to the more stringent LOA requirement (±5% of the range of the 5-min measurement) and smaller sample in the Shaffer et al. (2019) study.

**TABLE 4:** Minimum time period required to estimate 5-min HRV metrics adapted from Shaffer et al. (2019).

**Minimum UST period**	**HRV metric**
10 s	HR
60 s	pNN50, NN50, RMSSD, SDNN
90 s	TINN, LF absolute power, SD1, and SD2
120 s	HRV triangular index, DFA α1
180 s	LFnu, HF absolute power, HFnu, LF/HF power, DFA α2, DET, SampEn
240 s	ShanEn

*DFA α1, detrended fluctuation analysis, which describes short-term fluctuations; DFA α2, detrended fluctuation analysis, which describes long-term fluctuations; ECG, electrocardiogram; HF ms^2^, absolute power of the high frequency band; HF nu, relative power of the high frequency band in normal units; HF peak, highest amplitude frequency in the HF band; HF%, HF power as a percentage of total power; HR, heart rate; HTI, HRV triangular index or integral of the density of the NN interval histogram divided by its height; limits of agreement, criterion that two methods are equivalent if there is an acceptable *a priori* difference between their values in absolute units; LF ms^2^, absolute power of the low frequency band; LF nu, relative power of the low frequency band in normal units; LF peak, highest amplitude frequency in the LF band; LF%, LF power as a percentage of total power; LF/HF, ratio of LF-to-HF power; NN interval, time between adjacent normal heartbeats; nu, normal units calculated by dividing the absolute power for a specific frequency band by the summed absolute power of the LF and HF bands; pNN50, percentage of successive interbeat intervals that differ by more than 50 ms; RMSSD, root mean square of successive R–R interval differences; R–R interval, time between all adjacent heartbeats; SampEn, sample entropy, which measures signal regularity and complexity; SD1, Poincaré plot standard deviation perpendicular to the line of identity; SD2, Poincaré plot standard deviation along the line of identity; SD1/SD2, ratio of SD1 to SD2 that measures the unpredictability of the R–R time series and autonomic balance under appropriate monitoring conditions; SDNN, standard deviation of NN intervals; TINN, triangular interpolation of the R–R interval histogram or baseline width of the RR interval histogram; total power, sum of power (ms^2^) in VLF, LF, and HF bands; UST, ultra-short-term (<5 min).*

## Practical Recommendations

Recommendations for analyses of data from method-comparison studies differ. As previously mentioned, correlation/regression analyses quantify the degree of association between variables but do not denote agreement (Bland and Altman, 1986). As such, we recommend using LoA solutions to assess whether two methods produce comparable results. Although oft-cited guidelines recommend correlation/regression analyses in addition to the LoA solutions (Dewitte et al., 2002), most researchers incorrectly consider them to be supplemental (Dewitte et al., 2002; Bunce, 2009). Although correlation/regression analyses may answer certain questions that are relevant in method-comparison studies (e.g., whether two measures are *not* associated), there is a strong argument against their inclusion in favor of only reporting the LoA and their respective confidence intervals (Bland and Altman, 1986; Bunce, 2009). Prior to conducting method-comparison studies, researchers should consider whether conducting correlation/regression analyses is appropriate.

Assuming that researchers obtain 10-s, 20-s, 30-s, 60-s, 90-s, 120-s, and 180-s RMSSD values and want to determine the shortest period that can estimate a 300-s RMSSD measurement, they should consider the following steps:

(1) Determine whether the RMSSD measurements are normally distributed. If not, use a logarithmic transformation like log(e) or the natural log (ln).
(2) Determine *a priori* the largest acceptable difference between 30-s and 300-s RMSSD values.
(3) Prepare difference plots like Bland–Altman using a 95% confidence interval and then conduct an equality test (e.g., Student’s *t-*test) to confirm that the 30-s and 300-s RMSSD values are identical.
(4) If the 30-s RMSSD measurement passes the equality test, then a suitable surrogate has been found. If it fails the test, perform the same analysis with the 60-s measurement, and so on.

## Conclusion

Eight of the 11 HRV criterion validity studies we reviewed used correlational and/or group difference criteria that did not control for measurement bias. Because these criteria do not require a maximum acceptable difference (e.g., 5 bpm), they could yield an UST heart rate value that was 10 bpm higher or lower than its 5-min counterpart. Therefore, minimum recording length prescriptions from studies that used these criteria (Thong et al., 2003; Schroeder et al., 2004; McNames and Aboy, 2006; Salahuddin et al., 2007; Li et al., 2009; Nussinovitch et al., 2011; Brisinda et al., 2015) should be treated with caution and confirmed by studies that use a LOA criterion and confirmative equality tests. As Fleming and DeMets (1996) succinctly stated, “A correlate does not a surrogate make” (p. 605).

The routine use of UST HRV measurements in medicine, performance, and personal fitness assessment awaits advances in six key areas. First, HRV monitoring with automatic artifact correction needs to be added to existing hardware (e.g., activity trackers, pulse oximeters, and smartwatches). Second, researchers should identify the short-term HRV metrics (e.g., RMSSD) most strongly associated with health and performance outcomes. Third, researchers should determine the minimum UST time periods required to estimate these short-term HRV features with respect to age and sex. We recommend a LOA criterion based on the *a priori* determination of the largest acceptable difference between UST and short-term values confirmed by an equality test. Fourth, researchers should demonstrate that UST HRV metrics themselves can forecast real-world health or performance outcomes. UST measurements are proxies of proxies. They seek to replace short-term values, which, in turn, attempt to estimate reference standard long-term metrics. This criterion validity requirement is the most intractable and may prove insurmountable. Fifth, researchers should establish UST HRV norms stratified by age and sex. Sixth, researchers and manufacturers need to educate healthcare professionals and the public about what HRV means, its importance to their health and performance, how it should be measured, and the strategies that can increase it. These six breakthroughs are necessary before HRV monitoring can be more widely used in medicine, performance, and personal health care.

## Author Contributions

FS reviewed the literature, wrote the initial manuscript, and made subsequent revisions following feedback and editorial suggestions for all drafts from ZM and CZ. ZM reviewed the literature, created and managed the UST literature database, and summarized and critiqued the UST studies. CZ reviewed the method agreement literature and wrote the methodological critique section. All authors contributed to the article and approved the submitted version.

## Conflict of Interest

The authors declare that the research was conducted in the absence of any commercial or financial relationships that could be construed as a potential conflict of interest.
